# Effect of Pay-For-Outcomes and Encouraging New Providers on National Health Service Smoking Cessation Services in England: A Cluster Controlled Study

**DOI:** 10.1371/journal.pone.0123349

**Published:** 2015-04-15

**Authors:** Hugh McLeod, Deirdre Blissett, Steven Wyatt, Mohammed A Mohammed

**Affiliations:** 1 Health Economics Unit, School of Health and Population Sciences, University of Birmingham, Birmingham B15 2TT, UK; 2 NHS Midlands and Lancashire Commissioning Support Unit, Kingston House, High Street, West Bromwich B70 9LD, UK; 3 School of Health Studies, University of Bradford, Richmond Road, Bradford BD7 1DP, UK; The University of Auckland, NEW ZEALAND

## Abstract

**Background:**

Payment incentives are known to influence healthcare but little is known about the impact of paying directly for achieved outcomes. In England, novel purchasing (commissioning) of National Health Service (NHS) stop smoking services, which paid providers for quits achieved whilst encouraging new market entrants, was implemented in eight localities (primary care trusts (PCTs)) in April 2010. This study examines the impact of the novel commissioning on these services.

**Methods:**

Accredited providers were paid standard tariffs for each smoker who was supported to quit for four and 12 weeks. A cluster-controlled study design was used with the eight intervention PCTs (representing 2,138,947 adult population) matched with a control group of all other (n=64) PCTs with similar demographics which did not implement the novel commissioning arrangements. The primary outcome measure was changes in quits at four weeks between April 2009 and March 2013. A secondary outcome measure was the number of new market entrants within the group of the largest two providers at PCT-level.

**Results:**

The number of four-week quits per 1,000 adult population increased per year on average by 9.6% in the intervention PCTs compared to a decrease of 1.1% in the control PCTs (incident rate ratio 1∙108, p<0∙001, 95% CI 1∙059 to 1∙160). Eighty-five providers held ‘any qualified provider’ contracts for stop smoking services across the eight intervention PCTs in 2011/12, and 84% of the four-week quits were accounted for by the largest two providers at PCT-level. Three of these 10 providers were new market entrants. To the extent that the intervention incentivized providers to overstate quits in order to increase income, caution is appropriate when considering the findings.

**Conclusions:**

Novel commissioning to incentivize achievement of specific clinical outcomes and attract new service providers can increase the effectiveness and supply of NHS stop smoking services.

## Introduction

Smoking is a leading cause of premature mortality and morbidity in developed countries [1]. In 1998, the UK government published a strategy to reduce smoking in England, which led to the establishment of NHS smoking cessation support services [2]. Known as NHS stop smoking services, they comprise a range of interventions dominated by one-to-one counselling which provide smokers who want to quit with access to intensive behavioural support and prescribed pharmacotherapy [2]. NHS stop smoking services are effective and highly cost-effective [3–7]. However, their impact has been limited, with survey data indicating that only 6.2% of smokers trying to quit had used these services [8], despite smoking cessation having a high policy profile [9–10]. NHS stop smoking services have been commissioned at local health economy level, which until April 2013 were configured as 151 primary care trusts (PCTs) covering England. Commissioners set local targets for four-week quit rates, defined as the percentage of enrolled smokers reported to have quit at four weeks, which were subject to national monitoring arrangements if they were outside the range 35% to 70% [3]. Commissioners typically offered block contracts to providers meeting nationally defined criteria, which may use subcontractors to deliver services [3,4,11,12]. This approach to commissioning led to variation in the delivery of services and associated costs, and did not provide a strong incentive to maximise the number of quits achieved [2,12].

Payment incentives influence the behaviour of healthcare providers [13]. Policy makers in England have followed developments in the United States and introduced payment incentives relating to achievement of activity-based [14] and quality-based [15,16] measures. The UK government is seeking to further promote effectiveness by directly linking payments to outcomes, and literal ‘payment by results’ is being piloted for drug and alcohol misuse treatment [5] and in the wider public sector [17,18]. The move to pay-for-outcomes has implications for purchasers (commissioners), clinicians and provider organisations.

### Stop smoking services intervention

In April 2010, commissioners from eight PCTs in one region volunteered to collaborate with their host health authority (West Midlands Strategic Health Authority) and take a unified approach to piloting new contracts for stop smoking services. They adopted qualification-based provider regulations known as ‘any qualified provider’ regulations, which allowed any providers to deliver services that met specified criteria, including adhering to NHS service quality requirements and accepting new payment, contractual and reporting obligations [19,3]. This approach was intended to encourage new providers to enter the market and stimulate innovation. The commissioners also set outcome-based tariffs and the aim was to use the payment system to incentivize the achievement of quits. Different tariffs were introduced for target populations, such as those in routine and manual occupations and pregnant women. Payments were made for quits recorded at both four and 12 weeks, thereby incentivizing extended support for individuals attempting to stop smoking. A normative approach was taken by the commissioners to determining the tariffs shown in S1 Table [20]. In non-intervention PCTs, and the intervention PCTs before April 2010, stop smoking services providers were typically funded using block contracts, as described above [3,4,11,12]. The new tariffs were implemented across the eight participating PCTs for three years from April 2010 and our study examined progress made by the intervention over this period.

The objective of this study was to evaluate the impact of the stop smoking intervention and this study is the first to report the impact of commissioning NHS treatment using payment incentives based solely on achieving specific clinical outcomes combined with opening the provider market using qualification-based regulations [19].

## Methods

### Study design

We used a cluster controlled study design in which the eight intervention PCTs were retrospectively matched to a control group of the 64 PCTs with similar demographics which did not implement the outcome-based payment system. The Office for National Statistics (ONS) has categorised geographic areas of the UK based on similar local population characteristics using census data [21]. Each of the 151 PCTs in England have been assigned to one of 20 ONS subgroups, which are used by the Department of Health for comparing spend at programme level [22]. The eight intervention PCTs fall into six ONS subgroups along with 64 other PCTs for which the population characteristics are summarised in S1 Box. The adult population in 2012/13, defined as individuals aged 20 years and over, totalled 2,155,577 across the eight intervention PCTs and 17,582,642 across the 64 control PCTs. We used this ONS classification to determine the six clusters and associated control PCTs used in the analysis (Table 1). We used 2009/10 as the baseline and assessed performance over the subsequent three years.

**Table 1:** Intervention PCTs, ONS subgroups, clusters and number of control PCTs

Intervention PCT	Office for National Statistics subgroup	Cluster	Number of PCTs in the control group
A, B	Centres with Industry A	1	9
C	Centres with Industry B	2	7
D	Industrial Hinterlands A	3	14
E	Manufacturing Towns A	4	13
F	Prospering Smaller Towns B	5	12
G, H	Prospering Smaller Towns C	6	9

### Main outcome measures

Our outcome measures are 1) The number of enrolled smokers per 1,000 adult population. 2) The number of four-week quits per 1,000 adult population. A four-week quit is defined as a treated smoker self-reporting continuous abstinence from smoking from day 14 post-quit date whose quit status within 25 to 42 days of the quit date has been assessed (either face-to-face or by telephone, text, email or postal questionnaire) [10,23]. Two datasets on four-week quits are published; self-reported quits and self-reported quits verified by carbon monoxide (CO) testing. Neither dataset is ideal for assessing changes in quit performance; changes in CO-verified quits may reflect changes in CO verification activity rather than changes in the number of smokers quitting, and some self-reported quits are found to be invalid following CO testing. The most recently published national data for 2008/9 show that 15.3% (19,424/126,838) of self-reported quits by men and 16.5% (23,131/139,995) of self-reports quits by women were found to have a CO reading of more than 10ppm, and therefore were not deemed to be valid. In order to adjust for the over reporting of quits not confirmed by CO testing, we have excluded these gender specific proportions of quits not confirmed by CO testing. This assumption, which facilitates making use of both available published datasets on four-week quits, is investigated in our sensitivity analysis. 3) The number of four-week quits verified by CO testing as a percentage of enrolled smokers (the four-week quit rate used by the Department of Health to monitor performance). 4) The number of stop smoking services providers holding ‘any qualified provider’ contracts in 2011/12. 5) The percentage of four-week quits accounted for by the largest two providers at PCT-level in 2011/12, and whether they were existing or new market entrants. 6) The percentage of self-reported quits validated by CO testing over four years from April 2008 to March 2013.

### Data sources

Our main analysis used published PCT-level data on stop smoking services for the years 2009/10 to 2012/13 [24]. Data on the adult population were obtained from the ONS mid-year population estimates [25,26]. The provider-level data on four-week quits for the intervention in 2011/12 were provided by NHS West Midlands Healthcare Commissioning Services. These data are not directly comparable to the PCT-level data used in the rest of the analysis because of differences between definitions used for contractual and payment systems and those used for national reporting. In addition, in some intervention PCTs, there were parallel commissioning arrangements which supplemented the commissioned intervention scheme.

### Statistical Analysis

We began with exploratory plots of the outcome variables over time for each PCT nested within clusters. We used mixed effects Poisson regression models to account for “clustering” effects, with repeated measures nested within PCTs and PCTs nested within clusters (n = 6). PCTs and clusters (n = 6) had random intercept terms and PCTs had a random slope term. The response variable was a given smoking cessation outcome measure (e.g. the estimated number of four-week quits), with an exposure variable reflecting the area of opportunity (e.g. adult population). The model had three covariates—pilot PCT (yes/no), years (continuous, 0 to 3) and their interaction. The hypothesis of interest (i.e. "effect-size") was represented by the interaction term (rate of change of the incidence rate ratio). If the interaction term was significantly greater than 1 then we can conclude that the intervention PCTs have a higher rate of change than controls. Statistical significance was set at 5%. Plots were produced in the lattice package [27] in R [28] and modelling was undertaken in Stata 12 [29].

### Sensitivity analysis

We have undertaken sensitivity analysis at an aggregate level to explore whether changing our measure of quits or controls altered the findings. Our baseline analysis assumed that 16% of quits not confirmed by CO testing should be excluded, and we varied this by plus or minus 8%, (a) and (b) respectively, as well including all self-reported quits (c) and only CO-verified quits (d). We made three changes to the control. We reran the analysis taking account of the presence of the pre-existing ‘spearhead’ localities (e). The spearhead group was announced by the Department of Health in 2004, and comprised the 20% of local authorities in England facing the greatest health challenges [30]. The aim was to reduce health inequalities by targeting a range of interventions including smoking cessation services. Four of the intervention PCTs were in the spearhead group, and we compared these to those controls which were also spearhead PCTs. Similarly, we compared the four non-spearhead PCTs to only those control PCTs which were also non-spearhead PCTs. This reduced the total number of control PCTs from 64 to 48. We changed the control group in order to focus on non-intervention PCTs with the most similar performance to the intervention PCTs in terms of four-week quits per 1,000 adult population in the baseline year (2009/10). We included the two non-intervention PCTs with the most similar level of performance to each of the eight intervention PCTs (f). Using this criterion for selecting control PCTs, intervention PCTs D and E shared one control PCT and so the next closest performing non-intervention PCT was selected as a control PCT for PCT E. We also reran the analysis including all 143 non-intervention PCTs in England in the control group (g).

## Results

### Enrolment

**Fig 1:**
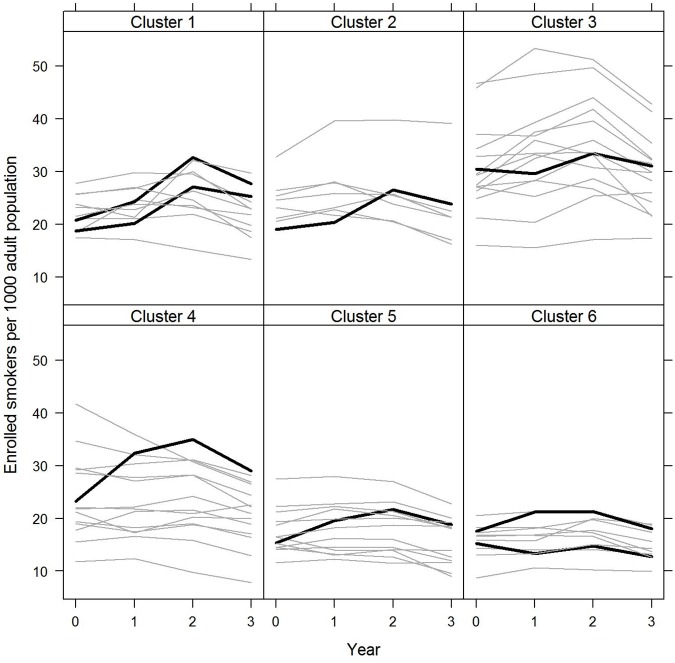
4-week quits per 1,000 adult population for intervention and control PCTs by cluster: 2009/10 to 2012/13 The figure shows the change in quits over time for the intervention PCTs compared to their comparators in each of the six clusters. The clusters use the Office for National Statistics (ONS) subgroup categories for geographic areas of the UK based on similar local population characteristics. The eight intervention PCTs fall into six ONS subgroups along with 64 other PCTs which form the controls. Intervention PCTs are shown in black and control PCTs are shown in grey. 2009/10 = 0 and 2012/13 = 3.

The intervention PCTs experienced an increase in the number of enrolled smokers per 1,000 adult population of 5.5% per year on average compared to a decrease of 2.5% per year in the control PCTs (Table 2; 5.5% = ((0.975*1.082)-1)*100). This difference, expressed as a ratio of rates (i.e. the interaction term) was statistically significant (1.082 = 1.055/0.975, p<0.001, 95% CI 1.040 to 1.127) (Table 2). The intervention PCTs’ baseline enrolment per 1,000 adult population was not significantly lower than that of the controls (0.955, p = 0.633, 95% CI 0.792 to 1.152) (Table 2). Cluster-level results are shown in S2 Table and the clusters exhibit a range of experience relating to enrolment over the four years (Fig 1). For example, the number of enrolled smokers per 1000 adult population for the intervention PCTs G and H and their nine control PCTs in cluster 6 (prospering smaller towns C) are comparatively similar and low in contrast to intervention PCT E and its 14 control PCTs in cluster 3 (industrial hinterlands A) (Fig 1 and S2 Table). In two of the intervention PCTs (D and H) progress with implementing the intervention was limited and some of the stop smoking services activity remained under pre-existing contractual arrangements.

**Table 2:** intervention and control PCTs, changes between 2009/10 and 2012/13: model findings

	incidence rate ratio	P	95% confidence interval
change in the number of individuals enrolled per 1,000 adult population			
intervention	0.955	0.633	0.792 to 1.152
year	0.975	<0.001	0.962 to 0.988
intervention.year	1.082	<0.001	1.040 to 1.127
constant	0.022	<0.001	0.019 to 0.027
change in the number of 4-week quits per 1,000 adult population			
intervention	0.869	0.061	0.751 to 1.007
year	0.989	0.161	0.974 to 1.004
intervention.year	1.108	<0.001	1.059 to 1.160
constant	0.010	<0.001	0.009 to 0.012
change in the number of CO-validated 4-week quits as a % of enrolled smokers			
intervention	1.060	0.661	0.816 to 1.377
year	1.028	0.006	1.008 to 1.049
intervention.year	1.039	0.204	0.979 to 1.102
constant	0.325	<0.001	0.292 to 0.361
change in the number of CO-validated 4-week quits as a percentage of all self-reported quits			
intervention	1.180	0.145	0.945 to 1.473
year	1.013	0.102	0.997 to 1.029
intervention.year	1.016	0.504	0.969 to 1.065
constant	0.673	<0.001	0.625 to 0.725
change in the number of smokers enrolled in stop smoking services not lost to follow-up per 1,000 adult population			
intervention	0.850	0.147	0.683 to 1.059
year	0.980	0.034	0.962 to 0.998
intervention.year	1.049	0.089	0.993 to 1.108
constant	0.017	<0.001	0.015 to 0.020
change in the number of 4-week quits per enrolled smokers not lost to follow-up			
intervention	1.016	0.800	0.896 to 1.153
year	1.009	0.099	0.998 to 1.020
intervention.year	1.055	0.001	1.022 to 1.090
constant	0.610	<0.001	0.585 to 0.636

### Four-week quits

The number of quits per 1,000 adult population increased by 9.6% per year on average in the intervention PCTs, compared with a decrease of 1.1% per year in the control PCTs, and the difference in the ratio of rates was statistically significant (incident rate ratio 1.108, p<0.001, 95% CI 1.059 to 1.160) (Table 2 and S3 Table).

The approximately tenfold better rate of improvement in performance of the intervention PCTs compared to the control PCTs was maintained when we varied the percentage of excluded quits not confirmed by CO testing by plus or minus 8%, as well including all self-reported quits and only CO-verified quits (S4 Table). The finding was similarly not affected by changing the control group to account for ‘spearhead’ localities or baseline quit performance, or comparison with all non-intervention PCTs in England (S4 Table). Data on quit numbers are shown in S5 Table.

**Fig 2:**
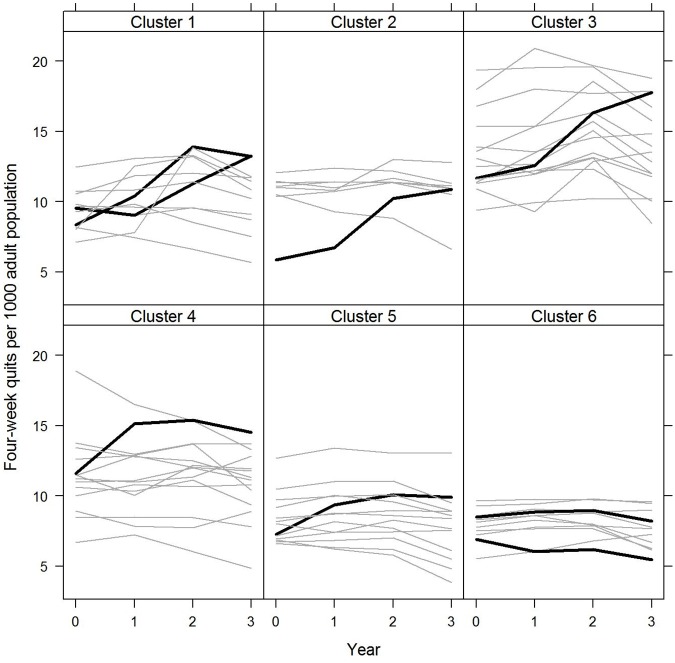
Enrolled smokers per 1,000 adult population for intervention and control PCTs by cluster: 2009/10 to 2012/13 The figure shows the change in this measure of quits over time for the intervention PCTs compared to their comparators in each of the six clusters. The clusters use the Office for National Statistics (ONS) subgroup categories for geographic areas of the UK based on similar local population characteristics. The eight intervention PCTs fall into six ONS subgroups along with 64 other PCTs which form the controls. Intervention PCTs are shown in black and control PCTs are shown in grey. 2009/10 = 0 and 2012/13 = 3.

The four-week quit rate used by the Department of Health to monitor performance, defined as the number of CO-validated four-week quits as a percentage of the number of enrolled smokers, increased by 6.8% per year on average in the intervention PCTs and 2.8% per year in the control PCTs, and the difference in change was not statistically significant (incident rate ratio 1.039, p = 0.204, 95% CI 0.979 to 1.102) (Table 2, S6 Table and Fig 2). By 2012/13, the CO-validated four-week quit rate was 42.8% in the intervention PCTs compared to 35.7% in the controls. The difference in change was similar when including all self-reported quits (S4 Table).

### Stop smoking service providers

In 2011/12, 85 providers held stop smoking services contracts in the intervention PCTs under the ‘any qualified provider’ regulations (Table 3). Six of these providers were existing NHS community services providers. The other providers were either existing providers of stop smoking services which had held subcontracts with the community services providers, or new entrants to the market. They included general practitioners, hospital-based clinician teams, and commercial and third-sector organisations.

**Table 3:** Number of providers and percentage of quits by intervention PCTs

Intervention PCT	number of intervention providers	self-reported 4-week quits in 2011/12^a^			
		number	% by largest intervention provider	% by second- largest intervention provider	% by all other intervention providers
A	10	1936	67	19	14
B	13	2874	73	14	14
C	22	2179	37	27	35
D	27	2869	42	16	42
E	6	1420	88	8	4
F	6	1865	90	6	4
G	19	3138	79	6	14
H	26	917	48	6	46
total	85	17198	75	9	16

^a^ Intervention data on ‘any qualified provider’ contracts

The intervention’s data on ‘any qualified provider’ contracts show that six providers were the largest PCT-level providers across the intervention, and they accounted for 75% of the total intervention quits in 2011/12 (Table 3). In five of the PCTs, these largest providers were four of the existing NHS community services providers. The largest provider in PCT D was also an existing provider. The largest provider in the other two PCTs [C,H] was a new entrant to the market. This provider was also the second-largest provider in four of the other intervention PCTs [A, B, E, G], and was the second-largest provider across all eight PCTs. Two of the other second-largest providers were new entrants (PCTs C and F) and the other two were existing providers (PCTs D and H), and these four providers accounted for 9% of the total intervention quits in 2011/12 (Table 3). The three PCTs with the largest number of providers were those in which the main provider accounted for a minority of quits [C, D, H].

### Carbon monoxide validation

A measure of the quality of smoking cessation services is the percentage of self-reported quits validated by CO testing, which increased by 3.0% per year on average in the intervention PCTs compared to 1.3% per year in the control PCTs. Although this difference was not statistically significant (incident rate ratio 1.016, p = 0.504, 95% CI 0.969 to 1.065) (Table 2 and S7 Table), the intervention PCTs had comparatively high validation levels throughout the period compared to controls in four of the clusters (S1 Fig). By 2012/13, 85.5% of self-reported quits were validated in the intervention PCTs compared to 69.7% in the controls. Data on CO-validated quits are shown in S5 Table.

### Intervention impact

As noted above, providers in the intervention PCTs increased reach to smokers by getting more individuals to set a quit date. However, they did not maintain contact with the larger group of individuals attempting to quit. The difference in change in the number of individuals not lost to follow-up per 1,000 adult population for intervention PCTs and control PCTs (2.8% and -2.0% per year, respectively) was not statistically significant (Table 2, S8 Table and S2 Fig). Instead, the providers appear to have worked with similar numbers of individuals over the four weeks following the setting of quit dates (defined as those not lost to follow-up), and achieved more quits at four weeks from this group; the difference in the increases of 6.5% per year on average for the intervention PCTs compared to 0.9% per year for the control PCTs was statistically significant (Table 2, S9 Table and S3 Fig). Whether intervention providers have, for example, implemented screening of individuals who have set quit dates, in order to better target their support to smokers thought likely to quit, is not known.

## Discussion

### Findings

The intervention PCTs that adopted novel commissioning policies, comprising pay-for-outcomes and opening the market to new providers, experienced substantial increases in quits compared to the controls: the number of four-week quits per 1,000 adult population increased by 9.6% per year on average in the intervention PCTs, compared with a decrease of 1.1% per year in the control PCTs. This finding is important because despite the growth in stop smoking services, which are viewed as cost-effective, the scale of these services remains small in the face of the challenge to reduce smoking prevalence [2]. The largest 10 providers in the intervention accounted for 84% of the four-week quits in 2011/12, and three of these providers were new market entrants. Although provision was dominated by existing NHS community services providers, the finding that a new entrant generated most quits in two of the eight intervention PCTs suggests that provider diversity has been promoted. Whilst intervention and control PCTs experienced increases in CO-validation rates over time, the intervention PCTs achieved comparatively better performance than the controls.

The wider adoption of the novel commissioning policies described in our study is being pursued in the NHS and other public sectors [17,18], despite very limited existing evidence on this type of incentive. A recent Cochrane review [31] assessed the effect of financial incentives on the quality of health care provided by primary care physicians. Only one of the seven studies included in the review was of a payment per patient achieving an outcome, which was offered as part of a multifaceted RCT to selected general practitioners in Germany for patients who had quit smoking [32]. The general practitioners were given training on promoting smoking cessation as part of their regular patient contacts, and this intervention was not found in isolation to reduce quits [32,33]. Other systematic reviews of pay-for-performance in healthcare settings similarly include a myriad of payment incentives across diverse services and contexts, and the evidence on effectiveness [16,34–37], and cost-effectiveness [36–38] is limited and mixed. These reviews include only one instance of pay-for-outcomes: an uncontrolled before-and-after pilot study of English general practitioners reported that a payment for patients who had stopped smoking was viewed as too low to warrant checking whether patients had stopped smoking [36,39,40]. Incentivizing behaviour by funding service provision entirely on the basis of outcomes achieved, rather than via a bonus supplementing a base payment, has been subject to very limited testing despite its high policy profile [17,18]. As far as we are aware, our study is the first to report the impact of commissioning NHS treatment funded solely on achieving specific clinical outcomes combined with using qualification-based provider regulations.

### Strengths and limitations of the study

**Fig 3:**
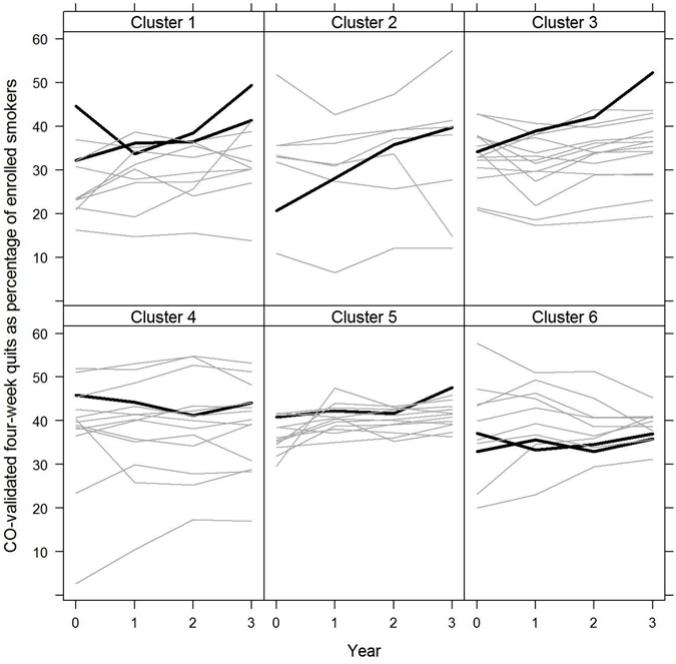
CO-validated 4-week quits as a percentage of the enrolled smokers for intervention and control PCTs by cluster: 2009/10 to 2012/13 The figure shows the change in enrolment over time for the intervention PCTs compared to their comparators in each of the six clusters. The clusters use the Office for National Statistics (ONS) subgroup categories for geographic areas of the UK based on similar local population characteristics. The eight intervention PCTs fall into six ONS subgroups along with 64 other PCTs which form the controls. Intervention PCTs are shown in black and control PCTs are shown in grey. 2009/10 = 0 and 2012/13 = 3.

Our cluster controlled study provides an appropriate quasi-experimental design for this type of policy intervention [41]. Our approach to selecting controls on the criterion of sharing local population characteristics, as defined by the ONS subgroup clusters, attempted to account for the different PCT-specific contexts in which the intervention took place (illustrated in the plots; Figs 1 and 3). For example, PCTs G and H were the least successful of the intervention PCTs in terms of increasing the number of enrolled smokers per 1000 adult population, but it is apparent from the experience of their nine control PCTs in cluster 6 (prospering smaller towns C) that PCT G maintained comparatively high enrolment numbers (Fig 1). However, the strong performance of the intervention PCTs was also evident when matching the intervention PCTs to the 16 non-intervention PCTs in England with the most similar baseline performance in terms of four-week quits per 1,000 adult population or comparing the intervention PCTs to all other PCTs in England (S4 Table f-g).

By adjusting the recorded quit data for the over-reporting of non-validated quits, we have attempted to improve the basis for making comparisons over time and between localities. As noted above, we did not use CO-validated quits only in our main analysis because changes in this measure may reflect changes in CO-verification activity rather than changes in the number of smokers quitting. Furthermore, although CO verification is viewed as an important quality indicator, and a comparatively high proportion of four-week quits in the intervention PCTs were validated by CO testing (86% in 2012/13), the test does not provide assurance that abstinence has occurred beyond a few hours, and certainly not since two weeks of the quit dates. To the extent that the intervention introduced a new incentive for providers to overstate four-week quits achieved in order to increase income, caution is appropriate when considering the findings. However, this incentive may have been moderated through the introduction of random audits of cases carried out by commissioners in the intervention PCTs, and this activity suggests that the outcome of the intervention should not be attributed to gaming or changes in record-keeping. The requirement to monitor data supplied by providers paid for successful quit data was recognised as being “especially important” in national guidance [3]. In addition, if providers systematically lied about self-reported quit status, it might be expected that CO-validation rates would have been comparatively low in the intervention PCTs. Furthermore, prior to the intervention, and in the control PCTs, where there tended to be a single or coordinating provider, a similar incentive to overstate four-week quits could have been driven by the obligation to hit locally agreed targets for four-week quit rates [42]. Nationally, PCTs were expected to achieve year-on-year increases in the number of four-week quits, and PCTs’ performance was subject to mandatory quarterly data reporting requirements and performance monitoring of four-week quit rates by strategic health authorities [42]. Overall, performance of stop smoking services had a high national profile, and all PCTs would have had good reason to fully report all quits [3,2]. However, the quit rate measure could potentially have be manipulated through the reporting of the data on the number of smokers enrolled in stop smoking services. A potential weakness of the data we have used on the number of smokers enrolled is that providers may have faced different incentives for recording these data. Providers operating in the intervention PCTs were required to record enrolment as smokers started to access stop smoking services. Before the intervention began, and in other localities, providers may have had little incentive to record enrolment until after the outcome was known at four weeks. This may have contributed to the larger increase in enrolment and loss to follow up experienced by the intervention PCTs.

Another measure of the reported impact of stop smoking services is the number of CO-validated four-week quits beyond those estimated to have occurred with only a prescription for smoking cessation medication and no behavioural support [2]. For the reasons noted above, we do not advocate using measures of performance which rely on CO-validated quits only and the number of enrolled smokers, but we report this measure of impact for the intervention in the supplementary information (S1 Text and S10 Table).

The intervention PCTs introduced enhanced payments for providers achieving quits in a range of target subpopulations, the assessment of which is constrained by limited relevant published data. Nevertheless, further analysis should explore whether the intervention was associated with ‘cream-skimming’ by providers seeking to avoid treating patients with more complex needs [19]. The intervention introduced an incentive to achieve quits at 12 weeks, and to the extent that control PCTs would not have shared this incentive, our analysis of performance at four weeks may understate the impact of the intervention.

Intervention PCT C in cluster 2 had a comparatively low baseline enrolment rate, and it could be argued that this made it comparatively easy to improve its performance. This may be so, but the majority of intervention PCTs were not in this situation, and it is also apparent that enrolment across the intervention PCTs and controls was typically very low in relation to the 20% of the adult population who smoke [43]. It is possible that some of our control PCTs also implemented locally agreed outcome-based payment systems, which could have resulted in our study underestimating the impact of the intervention. However, we checked the published tenders for stop smoking services which should be associated with such a change, and found no record of outcome-based payment systems being introduced in our controls [44]. We have not undertaken a cost-effectiveness analysis of the intervention because of the lack of comparable data. Nevertheless, based on the intervention’s tariff prices, and estimates on relapse rates to 12 months and associated gains to health-related quality of life, it is clear that the services provided in the intervention can be viewed as highly cost-effective (see S2 Text).

## Conclusion

Our findings suggest that the novel commissioning arrangements can improve the effectiveness and supply of the NHS stop smoking services. As the international policy momentum in payment-for-outcomes builds, this large-scale example of its application has relevance for policy makers and clinicians working in a range of healthcare settings and systems.

### Future research

Several aspects of the intervention merit further study. 1) The changes in quits, enrolment and follow-up suggest that practice has changed in the intervention PCTs, but the precise mechanisms associated with the change in financial incentives, remain unclear. 2) We have focused on aggregate results, and study of individual clusters may provide useful insight into implementation of the intervention in local contexts. 3) By introducing payment incentives to achieve 12-week quits, the intervention PCTs have started to explore the provision of longer term support for smokers beyond the four-week threshold used by the Department of Health. This is important because about three-quarters of smokers who have quit at four weeks will relapse within one year [45]. However, the impact on the relapse rate for those who have abstained from smoking at 12 weeks is not clear, although studies show that the majority of smokers attempting to stop smoking without treatment relapse within eight days of their quit date [46]. Given the objective to achieve long-term quits, the intervention PCTs’ experience warrants further evaluation.

## Supporting Information

S1 Box ONS subgroups and characteristics for intervention and control PCTs (DOCX)

S1 Table Standard Stop Smoking Service Tariffs introduced in the intervention PCTs (DOCX)

S2 Table Change in the number of individuals enrolled in stop smoking services per 1,000 adult population for intervention and control PCTs between 2009/10 and 2012/13: model findings (DOCX)

S3 Table Change in the number of 4-week quits per 1,000 adult population for intervention and control PCTs between 2009/10 and 2012/13: model findings (DOCX)

S4 Table Sensitivity analyses relating to all intervention and control PCTs: model findings (DOCX)

S5 Table Activity for intervention and control PCTs by cluster and year (DOCX)

S6 Table Change in the number of CO-validated 4-week quits as a percentage of enrolled smokers for intervention and control PCTs between 2009/10 and 2012/13: model findings (DOCX)

S7 Table Change in the number of smokers enrolled in stop smoking services not lost to follow-up per 1,000 adult population for intervention and control PCTs between 2009/10 and 2012/13: model findings (DOCX)

S8 Table Change in the number of 4-week quits per enrolled smokers not lost to follow-up for intervention and control PCTs between 2009/10 and 2012/13: model findings (DOCX)

S9 Table Change in the number of CO-validated 4-week quits as a percentage of all self-reported quits for intervention and control PCTs between 2009/10 and 2012/13: model findings (DOCX)

S10 Table Impact of stop smoking services in intervention and control PCTs (DOCX)

S1 Fig CO-validated 4-week quits as a percentage of self-reported 4-week quits for intervention PCTs and control by cluster: 2009/10 to 2012/13 (DOCX)

S2 Fig Smokers enrolled in stop smoking services not lost to follow-up per 1,000 adult population for intervention PCTs and control by cluster: 2009/10 to 2012/13 (DOCX)

S3 Fig 4-week quits as a percentage of enrolled smokers not lost to follow-up for intervention PCTs and control by cluster: 2009/10 to 2012/13 (DOCX)

S1 Text A measure of the impact of stop smoking services (DOCX)

S2 Text A note on cost-effectiveness (DOCX)
