# Supplementary material for: Effect of Pay-For-Outcomes and Encouraging New Providers on National Health Service Smoking Cessation Services in England: A Cluster Controlled Study
Source: PLoS One. 2015 Apr 15;10(4):e0123349. doi: 10.1371/journal.pone.0123349 (PMC4398496; doi:10.1371/journal.pone.0123349)
Supplement: S1 Box — (DOCX) [file pone.0123349.s001.docx]

**Supporting information**

**S1 Box ONS subgroups and characteristics for intervention and control PCTs**

| PCT | ONS subgroup | ONS subgroup characteristics, variables with a proportion | | |
| --- | --- | --- | --- | --- |
|  |  | far below the national average | close to the national average | far above the national average |
| A, B | Centres with Industry A | the percentage of people identifying themselves as Black African, Black Caribbean or Other Black | • People who were not born in the United Kingdom  • Pensioners who live alone  • People who rent their accommodation from the public sector  • People who are students  • People who are long-term unemployed  • People who work in health or social care. | • People who identify themselves as Indian, Pakistani, Bangladeshi or Other Asian  • People who live in a terraced house  • People who have no central heating  • People who work in manufacturing. |
| C | Centres with Industry B |  | • People aged between 25 and 44  • People who rent their accommodation from the private sector  • People who live in flats  • People who work in the hotel or catering industries  • People who work in health or social care. | • People who identify themselves as Indian, Pakistani, Bangladeshi or Other Asian  • People who identify themselves as Black African, Black Caribbean or Other Black  • People who live in lone parent households  • People who rent their accommodation from the public sector  • People who are students. |
| D | Industrial Hinterlands A | • People who identify themselves as Black African, Black Caribbean or Other Black  • People who were not born in the United Kingdom. | • Average household size  • Women who look after their home or family. | • People of working age with a limiting long-term illness  • People who provide unpaid care. |
| E | Manufacturing Towns - A | • People who identify themselves as Black African, Black Caribbean or Other Black  • People who were not born in the United Kingdom. | • People who rent their accommodation from the public sector  • People who are unemployed  • People who are long-term unemployed  • People who work in health or social care. |  |
| F | Prospering Smaller Towns B | • People who identify themselves as Indian, Pakistani, Bangladeshi or Other Asian  • People who identify themselves as Black African, Black Caribbean or Other Black  • People who are separated, widowed or divorced  • Average number of people per room  • People who take public transport to work. | • People who rent their accommodation privately  • People who work in a professional or managerial occupation  • People who work in a routine occupation  • Men who work part-time  • People who work in manufacturing  • People who work in the hotel or catering trades. |  |
| G, H | Prospering Smaller Towns C | • People who identify themselves as Black African, Black Caribbean or Other Black  • People who take public transport to work. | • Children aged four or under  • People aged between five and 14  • People aged between 25 and 44  • People with a higher education qualification  • People who work in a routine occupation  • People who provide unpaid care  • People who work in agriculture. | people who live in a two car household. |

Source: [21] ONS= Office for National Statistics
