# Supplementary material for: Effect of Pay-For-Outcomes and Encouraging New Providers on National Health Service Smoking Cessation Services in England: A Cluster Controlled Study
Source: PLoS One. 2015 Apr 15;10(4):e0123349. doi: 10.1371/journal.pone.0123349 (PMC4398496; doi:10.1371/journal.pone.0123349)
Supplement: S1 Table — (DOCX) [file pone.0123349.s002.docx]

**Supp****orting information**

S1 Table Standard Stop Smoking Service Tariffs introduced in the intervention PCTs

| Population | Payment for services that do not incur the  cost of prescribing (£) | | Payment for services that incur the  cost of prescribing (£) | |
| --- | --- | --- | --- | --- |
|  | 4-week quit | 12-week quit | 4-week quit | 12-week quit |
| General | 94 | 129 | 166 | 228 |
| Target | 136 | 271 | 214 | 427 |
