# Supplementary material for: Effect of Pay-For-Outcomes and Encouraging New Providers on National Health Service Smoking Cessation Services in England: A Cluster Controlled Study
Source: PLoS One. 2015 Apr 15;10(4):e0123349. doi: 10.1371/journal.pone.0123349 (PMC4398496; doi:10.1371/journal.pone.0123349)
Supplement: S2 Table — (DOCX) [file pone.0123349.s003.docx]

**Supp****orting information**

**S2 Table Change in the number of individuals enrolled in stop smoking services per 1,000 adult population for intervention and control PCTs between 2009/10 and 2012/13: model findings**

|  |  | incidence rate ratio | P | 95% confidence interval |
| --- | --- | --- | --- | --- |
| all intervention and control PCTs | | | | |
|  | intervention | 0.955 | 0.633 | 0.792 to 1.152 |
|  | year | 0.975 | <0.001 | 0.962 to 0.988 |
|  | intervention.year | 1.082 | <0.001 | 1.040 to 1.127 |
|  | constant | 0.022 | <0.001 | 0.019 to 0.027 |
| cluster 1 | | | | |
|  | intervention | 0.872 | 0.206 | 0.706 to 1.078 |
|  | year | 0.982 | 0.317 | 0.946 to 1.018 |
|  | intervention.year | 1.141 | 0.003 | 1.047 to 1.243 |
|  | constant | 0.024 | <0.001 | 0.021 to 0.026 |
| cluster 2 | | | | |
|  | intervention | 0.746 | 0.046 | 0.560 to 1.174 |
|  | year | 0.962 | 0.026 | 0.929 to 1.027 |
|  | intervention.year | 1.140 | 0.008 | 1.034 to 1.256 |
|  | constant | 0.026 | <0.001 | 0.024 to 0.029 |
| cluster 3 | | | | |
|  | intervention | 0.978 | 0.939 | 0.549 to1.740 |
|  | year | 1.000 | 0.960 | 0.979 to 1.023 |
|  | intervention.year | 1.018 | 0.685 | 0.935 to 1.107 |
|  | constant | 0.031 | <0.001 | 0.027 to 0.036 |
| cluster 4 | | | | |
|  | intervention | 1.166 | 0.629 | 0.625 to 2.178 |
|  | year | 0.954 | 0.001 | 0.929 to 0.978 |
|  | intervention.year | 1.120 | 0.026 | 1.014 to 1.237 |
|  | constant | 0.023 | <0.001 | 0.020 to 0.027 |
| cluster 5 | | | | |
|  | intervention | 0.958 | 0.854 | 0.604 to 1.518 |
|  | year | 0.958 | 0.003 | 0.931 to 0.985 |
|  | intervention.year | 1.116 | 0.038 | 1.006 to 1.237 |
|  | constant | 0.018 | <0.001 | 0.016 to 0.020 |
| cluster 6 | | | | |
|  | intervention | 1.074 | 0.666 | 0.776 to 1.488 |
|  | year | 0.991 | 0.560 | 0.960 to 1.022 |
|  | intervention.year | 0.991 | 0.806 | 0.921 to 1.066 |
|  | constant | 0.016 | <0.001 | 0.014 to 0.018 |
