# Supplementary material for: Effect of Pay-For-Outcomes and Encouraging New Providers on National Health Service Smoking Cessation Services in England: A Cluster Controlled Study
Source: PLoS One. 2015 Apr 15;10(4):e0123349. doi: 10.1371/journal.pone.0123349 (PMC4398496; doi:10.1371/journal.pone.0123349)
Supplement: S3 Table — (DOCX) [file pone.0123349.s004.docx]

**Supp****orting information**

**S3 Table Change in the number of 4-week quits per 1,000 adult population for intervention and control PCTs between 2009/10 and 2012/13: model findings**

|  |  | incidence rate ratio | P | 95% confidence interval |
| --- | --- | --- | --- | --- |
| all intervention and control PCTs | | | | |
|  | intervention | 0.869 | 0.061 | 0.751 to 1.007 |
|  | year | 0.989 | 0.161 | 0.974 to 1.004 |
|  | intervention.year | 1.108 | <0.001 | 1.059 to 1.160 |
|  | constant | 0.010 | <0.001 | 0.009 to 0.012 |
| cluster 1 | | | | |
|  | intervention | 0.900 | 0.332 | 0.729 to 1.113 |
|  | year | 1.003 | 0.896 | 0.954 to 1.056 |
|  | intervention.year | 1.148 | 0.023 | 1.019 to 1.293 |
|  | constant | 0.010 | <0.001 | 0.009 to 0.011 |
| cluster 2 | | | | |
|  | intervention | 0.521 | <0.001 | 0.468 to 0.579 |
|  | year | 0.985 | 0.467 | 0.946 to 1.026 |
|  | intervention.year | 1.269 | <0.001 | 1.132 to 1.423 |
|  | constant | 0.011 | <0.001 | 0.011 to 0.012 |
| cluster 3 | | | | |
|  | intervention | 0.841 | 0.401 | 0.562 to 1.259 |
|  | year | 1.008 | 0.415 | 0.989 to 1.028 |
|  | intervention.year | 1.155 | <0.001 | 1.073 to 1.244 |
|  | constant | 0.014 | <0.001 | 0.012 to 0.015 |
| cluster 4 | | | | |
|  | intervention | 1.150 | 0.556 | 0.721 to 1.834 |
|  | year | 0.980 | 0.134 | 0.954 to 1.006 |
|  | intervention.year | 1.087 | 0.097 | 0.985 to 1.200 |
|  | constant | 0.011 | <0.001 | 0.010 to 0.013 |
| cluster 5 | | | | |
|  | intervention | 0.927 | 0.696 | 0.634 to 1.356 |
|  | year | 0.968 | 0.024 | 0.942 to 0.996 |
|  | intervention.year | 1.133 | 0.015 | 1.025 to 1.254 |
|  | constant | 0.009 | <0.001 | 0.008 to 0.009 |
| cluster 6 | | | | |
|  | intervention | 0.942 | 0.602 | 0.754 to 1.178 |
|  | year | 0.990 | 0.510 | 0.963 to 1.019 |
|  | intervention.year | 0.972 | 0.405 | 0.909 to 1.039 |
|  | constant | 0.008 | <0.001 | 0.007 to 0.009 |
