# Supplementary material for: Effect of Pay-For-Outcomes and Encouraging New Providers on National Health Service Smoking Cessation Services in England: A Cluster Controlled Study
Source: PLoS One. 2015 Apr 15;10(4):e0123349. doi: 10.1371/journal.pone.0123349 (PMC4398496; doi:10.1371/journal.pone.0123349)
Supplement: S4 Table — (DOCX) [file pone.0123349.s005.docx]

**Supp****orting information**

**S4 Table Sensitivity analyses relating to all intervention and control PCTs: model findings**

|  |  | incidence rate ratio | | P | | 95% confidence interval | |  |
| --- | --- | --- | --- | --- | --- | --- | --- | --- |
| change in 4-week quits excluding 24% of quits not confirmed by CO testing per 1,000 adult population | | | | | | | |  |
|  | intervention | 0.878 | | 0.082 | | 0.759 to 1.017 | |  |
|  | year | 0.990 | | 0.184 | | 0.975 to 1.005 | |  |
|  | Intervention.year | 1.110 | | <0.001 | | 1.061 to 1.161 | |  |
|  | constant | 0.010 | | <0.001 | | 0.009 to 0.011 | |  |
| change in 4-week quits excluding 8% of quits not confirmed by CO testing per 1,000 adult population | | | | | | | |  |
|  | intervention | 0.861 | | 0.064 | | 0.743 to 0.998 | |  |
|  | year | 0.989 | | 0.030 | | 0.974 to 1.004 | |  |
|  | Intervention.year | 1.107 | | 0.001 | | 1.058 to 1.158 | |  |
|  | constant | 0.011 | | <0.001 | | 0.009 to 0.012 | |  |
| change in all reported 4-week quits per 1,000 adult population | | | | | | | |  |
|  | intervention | 0.853 | | 0.036 | | 0.735 to 0.990 | |  |
|  | year | 0.988 | | 0.128 | | 0.974 to 1.003 | |  |
|  | Intervention.year | 1.105 | | <0.001 | | 1.056 to 1.156 | |  |
|  | constant | 0.011 | | <0.001 | | 0.009 to 0.012 | |  |
| change in all 4-week quits confirmed by CO testing per 1,000 adult population | | | | | | | | |
|  | intervention | | 1.015 | | 0.909 | | 0.792 to 1.300 | |
|  | year | | 1.001 | | 0.907 | | 0.980 to 1.023 | |
|  | Intervention.year | | 1.123 | | <0.001 | | 1.053 to 1.199 | |
|  | constant | | 0.007 | | <0.001 | | 0.006 to 0.008 | |
| change in 4-week quits per 1,000 adult population: the four ‘spearhead’ intervention PCTs compared to only those controls which were also ‘spearhead’ PCTs and the four non-spearhead intervention PCTs compared to only those control PCTs which were also non-spearhead PCTs | | | | | | | | |
|  | intervention | | 0.877 | | 0.063 | | 0.763 to 1.007 | |
|  | year | | 0.993 | | 0.446 | | 0.976 to 1.011 | |
|  | Intervention.year | | 1.104 | | <0.001 | | 1.054 to 1.156 | |
|  | constant | | 0.010 | | <0.001 | | 0.009 to 0.012 | |
| change in 4-week quits per 1,000 adult population: intervention PCTs compared to 16 control PCTs with similar performance in 2009/10 | | | | | | | | |
|  | intervention | | 0.991 | | 0.742 | | 0.942 to 1.044 | |
|  | year | | 0.999 | | 0.936 | | 0.966 to 1.032 | |
|  | Intervention.year | | 1.098 | | 0.001 | | 1.037 to 1.163 | |
|  | constant | | 0.009 | | <0.001 | | 0.008 to 0.010 | |
| change in 4-week quits per 1,000 adult population: intervention PCTs compared to all other PCTs in England | | | | | | | | |
|  | intervention | | 0.930 | | 0.502 | | 0.753 to 1.149 | |
|  | year | | 0.992 | | 0.094 | | 0.982 to 1.001 | |
|  | Intervention.year | | 1.106 | | <0.001 | | 1.060 to 1.154 | |
|  | constant | | 0.009 | | <0.001 | | 0.009 to 0.010 | |
| change in the number of all self-reported 4-week quits as a percentage of enrolled smokers | | | | | | | | |
|  | intervention | | 0.892 | | 0.027 | | 0.806 to 0.987 | |
|  | year | | 1.015 | | 0.005 | | 1.004 to 1.025 | |
|  | Intervention.year | | 1.021 | | 0.170 | | 0.991 to 1.053 | |
|  | constant | | 0.484 | | <0.001 | | 0.458 to 0.511 | |
