# Supplementary material for: Effect of Pay-For-Outcomes and Encouraging New Providers on National Health Service Smoking Cessation Services in England: A Cluster Controlled Study
Source: PLoS One. 2015 Apr 15;10(4):e0123349. doi: 10.1371/journal.pone.0123349 (PMC4398496; doi:10.1371/journal.pone.0123349)
Supplement: S5 Table — (DOCX) [file pone.0123349.s006.docx]

**Supp****orting information**

**S5 Table Activity for intervention and control PCTs by cluster and year**

|  | cluster | 2009/10 | | 2010/11 | | 2011/12 | | 2012/13 | |
| --- | --- | --- | --- | --- | --- | --- | --- | --- | --- |
|  |  | Inter-vention PCTs | control PCTs | Inter-vention PCTs | control PCTs | Inter-vention PCTs | control PCTs | Inter-vention PCTs | control PCTs |
| 4-week quits | 1 | 3776 | 17985 | 4182 | 19695 | 5516 | 20162 | 5822 | 17857 |
|  | 2 | 1295 | 18658 | 1508 | 18726 | 2320 | 19977 | 2476 | 18902 |
|  | 3 | 2231 | 31449 | 2425 | 32691 | 3165 | 35397 | 3466 | 31382 |
|  | 4 | 1410 | 33549 | 1852 | 33255 | 1896 | 33986 | 1801 | 32234 |
|  | 5 | 1693 | 36223 | 2206 | 37810 | 2403 | 38060 | 2378 | 34105 |
|  | 6 | 7021 | 31504 | 6874 | 33144 | 7050 | 32955 | 6416 | 30052 |
|  | total | 17425 | 169369 | 19047 | 175323 | 22351 | 180537 | 22359 | 164531 |
| Individuals not lost to follow-up | 1 | 6600 | 29965 | 6605 | 34040 | 8457 | 34652 | 7827 | 29750 |
|  | 2 | 2778 | 32583 | 2138 | 36766 | 2673 | 34905 | 2791 | 32705 |
|  | 3 | 3641 | 50886 | 3689 | 56756 | 3793 | 59481 | 4006 | 50249 |
|  | 4 | 1935 | 53551 | 2678 | 53211 | 2745 | 53517 | 2759 | 48159 |
|  | 5 | 2405 | 57156 | 2859 | 59927 | 3273 | 60330 | 3245 | 52675 |
|  | 6 | 11347 | 52400 | 10759 | 54183 | 10807 | 55039 | 9831 | 49837 |
|  | total | 28706 | 276541 | 28728 | 294883 | 31748 | 297924 | 30459 | 263375 |
| Individuals lost to  follow-up | 1 | 1793 | 12597 | 2980 | 11351 | 4609 | 12505 | 3869 | 9934 |
|  | 2 | 1425 | 10653 | 2430 | 11387 | 3322 | 12323 | 2637 | 9906 |
|  | 3 | 2184 | 17648 | 2011 | 19214 | 2684 | 20472 | 2045 | 17065 |
|  | 4 | 891 | 15576 | 1281 | 15385 | 1566 | 15679 | 838 | 12390 |
|  | 5 | 1170 | 18177 | 1744 | 17582 | 1910 | 17045 | 1284 | 15403 |
|  | 6 | 3588 | 10479 | 5216 | 10282 | 5992 | 13085 | 4555 | 9495 |
|  | total | 11051 | 85130 | 15662 | 85201 | 20083 | 91109 | 15228 | 74193 |
| Carbon monoxide validated 4-week quits | 1 | 3156 | 10593 | 3365 | 13053 | 4870 | 13575 | 5247 | 11980 |
|  | 2 | 868 | 12432 | 1282 | 12382 | 2145 | 13530 | 2157 | 11837 |
|  | 3 | 1986 | 23470 | 2217 | 23503 | 2727 | 26840 | 3165 | 23903 |
|  | 4 | 1296 | 24464 | 1752 | 24026 | 1778 | 24906 | 1588 | 21969 |
|  | 5 | 1459 | 27574 | 1947 | 31224 | 2159 | 30987 | 2155 | 27752 |
|  | 6 | 5269 | 23279 | 5448 | 25741 | 5703 | 25819 | 5258 | 23078 |
|  | total | 14034 | 121812 | 16011 | 129929 | 19382 | 135657 | 19570 | 120519 |
