# Supplementary material for: Effect of Pay-For-Outcomes and Encouraging New Providers on National Health Service Smoking Cessation Services in England: A Cluster Controlled Study
Source: PLoS One. 2015 Apr 15;10(4):e0123349. doi: 10.1371/journal.pone.0123349 (PMC4398496; doi:10.1371/journal.pone.0123349)
Supplement: S6 Table — (DOCX) [file pone.0123349.s007.docx]

**Supp****orting information**

**S6 Table Change in the number of CO-validated 4-week quits as a percentage of enrolled smokers for intervention and control PCTs between 2009/10 and 2012/13: model findings**

|  |  | incidence rate ratio | P | 95% confidence interval |
| --- | --- | --- | --- | --- |
| all intervention and control PCTs | | | | |
|  | intervention | 1.060 | 0.661 | 0.816 to 1.377 |
|  | year | 1.028 | 0.006 | 1.008 to 1.049 |
|  | intervention.year | 1.039 | 0.204 | 0.979 to 1.102 |
|  | constant | 0.325 | <0.001 | 0.292 to 0.361 |
| cluster 1 | | | | |
|  | intervention | 1.383 | 0.075 | 0.968 to 1.976 |
|  | year | 1.059 | 0.022 | 1.008 to 1.113 |
|  | intervention.year | 1.008 | 0.887 | 0.899 to 1.131 |
|  | constant | 0.254 | <0.001 | 0.218 to 0.295 |
| cluster 2 | | | | |
|  | intervention | 0.763 | 0.591 | 0.284 to 2.049 |
|  | year | 1.012 | 0.694 | 0.955 to 1.071 |
|  | intervention.year | 1.218 | 0.017 | 1.036 to 1.432 |
|  | constant | 0.291 | <0.001 | 0.205 to 0.412 |
| cluster 3 | | | | |
|  | intervention | 1.073 | 0.747 | 0.701 to1.641 |
|  | year | 1.016 | 0.051 | 1.000 to 1.033 |
|  | intervention.year | 1.131 | <0.001 | 1.064 to 1.202 |
|  | constant | 0.313 | <0.001 | 0.280 to 0.349 |
| cluster 4 | | | | |
|  | intervention | 1.314 | 0.628 | 0.436 to 3.962 |
|  | year | 1.035 | 0.313 | 0.968 to 1.108 |
|  | intervention.year | 0.948 | 0.681 | 0.736 to 1.222 |
|  | constant | 0.342 | <0.001 | 0.255 to 0.459 |
| cluster 5 | | | | |
|  | intervention | 1.066 | 0.338 | 0.935 to 1.216 |
|  | year | 1.033 | <0.001 | 1.015 to 1.052 |
|  | intervention.year | 0.014 | 0.681 | 0.950 to 1.081 |
|  | constant | 0.375 | <0.001 | 0.362 to 0.389 |
| cluster 6 | | | | |
|  | intervention | 0.905 | 0.462 | 0.596 to 1.375 |
|  | year | 1.013 | 0.595 | 0.965 to 1.065 |
|  | intervention.year | 0.997 | 0.963 | 0.888 to 1.119 |
|  | constant | 0.378 | <0.001 | 0.316 to 0.452 |
