# Supplementary material for: Effect of Pay-For-Outcomes and Encouraging New Providers on National Health Service Smoking Cessation Services in England: A Cluster Controlled Study
Source: PLoS One. 2015 Apr 15;10(4):e0123349. doi: 10.1371/journal.pone.0123349 (PMC4398496; doi:10.1371/journal.pone.0123349)
Supplement: S7 Table — (DOCX) [file pone.0123349.s008.docx]

**Supp****orting information**

**S7 Table Change in the number of smokers enrolled in stop smoking services not lost to follow-up per 1,000 adult population for intervention and control PCTs between 2009/10 and 2012/13: model findings**

|  |  | incidence rate ratio | P | 95% confidence interval |
| --- | --- | --- | --- | --- |
| All intervention and control PCTs | | | | |
|  | intervention | 0.850 | 0.147 | 0.683 to 1.059 |
|  | year | 0.980 | 0.034 | 0.962 to 0.998 |
|  | intervention.year | 1.049 | 0.089 | 0.993 to 1.108 |
|  | constant | 0.017 | <0.001 | 0.015 to 0.020 |
| cluster 1 | | | | |
|  | intervention | 0.945 | 0.748 | 0.669 to 1.334 |
|  | year | 1.002 | 0.964 | 0.927 to 1.083 |
|  | intervention.year | 1.063 | 0.515 | 0.885 to 1.276 |
|  | constant | 0.016 | <0.001 | 0.014 to 0.019 |
| cluster 2 | | | | |
|  | intervention | 0.600 | 0.083 | 0.337 to 1.069 |
|  | year | 0.966 | 0.267 | 0.909 to 1.027 |
|  | intervention.year | 1.048 | 0.592 | 0.883 to 1.244 |
|  | constant | 0.019 | <0.001 | 0.015 to 0.023 |
| cluster 3 | | | | |
|  | intervention | 0.827 | 0.569 | 0.430 to 1.590 |
|  | year | 1.005 | 0.728 | 0.979 to 1.030 |
|  | intervention.year | 1.021 | 0.675 | 0.926 to 1.127 |
|  | constant | 0.023 | <0.001 | 0.019 to 0.027 |
| cluster 4 | | | | |
|  | intervention | 0.995 | 0.989 | 0.502 to 1.975 |
|  | year | 0.960 | 0.002 | 0.936 to 0.985 |
|  | intervention.year | 1.144 | 0.006 | 1.038 to 1.260 |
|  | constant | 0.018 | <0.001 | 0.015 to 0.021 |
| cluster 5 | | | | |
|  | intervention | 0.782 | 0.292 | 0.495 to 1.236 |
|  | year | 0.956 | 0.001 | 0.930 to 0.983 |
|  | intervention.year | 1.143 | 0.008 | 1.036 to 1.262 |
|  | constant | 0.014 | <0.001 | 0.012 to 0.016 |
| cluster 6 | | | | |
|  | intervention | 0.932 | 0.707 | 0.647 to 1.343 |
|  | year | 0.995 | 0.796 | 0.956 to 1.035 |
|  | intervention.year | 0.946 | 0.245 | 0.861 to 1.039 |
|  | constant | 0.013 | <0.001 | 0.011 to 0.015 |
