# Supplementary material for: Effect of Pay-For-Outcomes and Encouraging New Providers on National Health Service Smoking Cessation Services in England: A Cluster Controlled Study
Source: PLoS One. 2015 Apr 15;10(4):e0123349. doi: 10.1371/journal.pone.0123349 (PMC4398496; doi:10.1371/journal.pone.0123349)
Supplement: S8 Table — (DOCX) [file pone.0123349.s009.docx]

**Supp****orting information**

**S8 Table Change in the number of 4-week quits per enrolled smokers not lost to follow-up for intervention and control PCTs between 2009/10 and 2012/13: model findings**

|  |  | incidence rate ratio | P | 95% confidence interval |
| --- | --- | --- | --- | --- |
| all intervention and control PCTs | | | | |
|  | intervention | 1.016 | 0.800 | 0.896 to 1.153 |
|  | year | 1.009 | 0.099 | 0.998 to 1.020 |
|  | intervention.year | 1.055 | 0.001 | 1.022 to 1.090 |
|  | constant | 0.610 | <0.001 | 0.585 to 0.636 |
| cluster 1 | | | | |
|  | intervention | 0.949 | 0.612 | 0.773 to 1.164 |
|  | year | 1.001 | 0.963 | 0.969 to 1.033 |
|  | intervention.year | 1.083 | 0.035 | 1.006 to 1.167 |
|  | constant | 0.600 | <0.001 | 0.550 to 0.655 |
| cluster 2 | | | | |
|  | intervention | 0.885 | 0.658 | 0.516 to 1.519 |
|  | year | 1.020 | 0.349 | 0.978 to 1.064 |
|  | intervention.year | 1.197 | 0.003 | 1.062 to 1.348 |
|  | constant | 0.594 | <0.001 | 0.491 to 0.719 |
| cluster 3 | | | | |
|  | intervention | 1.020 | 0.916 | 0.708 to 1.470 |
|  | year | 1.004 | 0.581 | 0.991 to 1.017 |
|  | intervention.year | 1.130 | <0.001 | 1.075 to 1.188 |
|  | constant | 0.597 | <0.001 | 0.543 to 0.656 |
| cluster 4 | | | | |
|  | intervention | 1.160 | 0.394 | 0.825 to 1.631 |
|  | year | 1.021 | 0.030 | 1.002 to 1.040 |
|  | intervention.year | 0.948 | 0.134 | 0.885 to 1.016 |
|  | constant | 0.625 | <0.001 | 0.571 to 0.685 |
| cluster 5 | | | | |
|  | intervention | 1.184 | 0.236 | 0.895 to 1.566 |
|  | year | 1.012 | 0.179 | 0.994 to 1.031 |
|  | intervention.year | 0.992 | 0.811 | 0.929 to 1.059 |
|  | constant | 0.618 | <0.001 | 0.572 to 0.667 |
| cluster 6 | | | | |
|  | intervention | 1.014 | 0.881 | 0.851 to 1.207 |
|  | year | 0.997 | 0.702 | 0.980 to 1.034 |
|  | intervention.year | 1.026 | 0.205 | 0.986 to 1.068 |
|  | constant | 0.621 | <0.001 | 0.576 to 0.669 |
