# Supplementary material for: Effect of Pay-For-Outcomes and Encouraging New Providers on National Health Service Smoking Cessation Services in England: A Cluster Controlled Study
Source: PLoS One. 2015 Apr 15;10(4):e0123349. doi: 10.1371/journal.pone.0123349 (PMC4398496; doi:10.1371/journal.pone.0123349)
Supplement: S9 Table — (DOCX) [file pone.0123349.s010.docx]

**Supp****orting information**

**S9 Table Change in the number of CO-validated 4-week quits as a percentage of all self-reported quits for intervention and control PCTs between 2009/10 and 2012/13: model findings**

|  |  | incidence rate ratio | P | 95% confidence interval |
| --- | --- | --- | --- | --- |
| all intervention and control PCTs | | | | |
|  | intervention | 1.180 | 0.145 | 0.945 to 1.473 |
|  | year | 1.013 | 0.102 | 0.997 to 1.029 |
|  | intervention.year | 1.016 | 0.504 | 0.969 to 1.065 |
|  | constant | 0.673 | <0.001 | 0.625 to 0.725 |
| cluster 1 | | | | |
|  | intervention | 1.390 | 0.093 | 0.947 to 2.040 |
|  | year | 1.038 | 0.001 | 1.015 to 1.061 |
|  | intervention.year | 1.003 | 0.915 | 0.954 to 1.054 |
|  | constant | 0.567 | <0.001 | 0.481 to 0.668 |
| cluster 2 | | | | |
|  | intervention | 1.112 | 0.794 | 0.502 to 2.464 |
|  | year | 0.984 | 0.476 | 0.942 to 1.028 |
|  | intervention.year | 1.101 | 0.129 | 0.972 to 1.247 |
|  | constant | 0.641 | 0.002 | 0.484 to 0.850 |
| cluster 3 | | | | |
|  | intervention | 1.287 | 0.123 | 0.934 to 1.775 |
|  | year | 1.009 | 0.305 | 0.991 to 1.028 |
|  | intervention.year | 0.994 | 0.864 | 0.929 to 1.063 |
|  | constant | 0.677 | <0.001 | 0.623 to 0.736 |
| cluster 4 | | | | |
|  | intervention | 1.374 | 0.519 | 0.522 to 3.615 |
|  | year | 1.007 | 0.813 | 0.951 to 1.066 |
|  | intervention.year | 0.976 | 0.824 | 0.789 to 1.207 |
|  | constant | 0.679 | 0.003 | 0.524 to 0.879 |
| cluster 5 | | | | |
|  | intervention | 1.123 | 0.410 | 0.852 to 1.478 |
|  | year | 1.025 | 0.023 | 1.003 to 1.047 |
|  | intervention.year | 0.994 | 0.884 | 0.920 to 1.074 |
|  | constant | 0.752 | <0.001 | 0.697 to 0.812 |
| cluster 6 | | | | |
|  | intervention | 1.032 | 0.858 | 0.732 to 1.454 |
|  | year | 1.014 | 0.567 | 0.968 to 1.062 |
|  | intervention.year | 1.021 | 0.706 | 0.916 to 1.139 |
|  | constant | 0.700 | <0.001 | 0.605 to 0.810 |
