# Supplementary material for: Effect of Pay-For-Outcomes and Encouraging New Providers on National Health Service Smoking Cessation Services in England: A Cluster Controlled Study
Source: PLoS One. 2015 Apr 15;10(4):e0123349. doi: 10.1371/journal.pone.0123349 (PMC4398496; doi:10.1371/journal.pone.0123349)
Supplement: S10 Table — (DOCX) [file pone.0123349.s011.docx]

**Supp****orting information**

**S10 Table Impact of stop smoking services in intervention and control PCTs**

| year | Number of CO-verified quits per 100,000 adult population | | | | | |
| --- | --- | --- | --- | --- | --- | --- |
|  | quits in excess of  the 25% level | | quits shortfall below  the 25% level | | quits attributed to the stop smoking services (quits above the 25% level minus quits below the 25% level) | |
|  | Intervention PCTs | Control PCTs | Intervention PCTs | Control PCTs | Intervention PCTs | Control PCTs |
| 2009/10 | 208 | 214 | 9 | 31 | 199 | 183 |
| 2010/11 | 237 | 234 | 0 | 32 | 237 | 202 |
| 2011/12 | 300 | 240 | 0 | 20 | 300 | 220 |
| 2012/13 | 378 | 225 | 0 | 20 | 378 | 205 |
