# Supplementary material for: Effect of Pay-For-Outcomes and Encouraging New Providers on National Health Service Smoking Cessation Services in England: A Cluster Controlled Study
Source: PLoS One. 2015 Apr 15;10(4):e0123349. doi: 10.1371/journal.pone.0123349 (PMC4398496; doi:10.1371/journal.pone.0123349)

**Supp****orting information**

**S1 Figure Smokers enrolled in stop smoking services not lost to follow-up per 1,000 adult population for intervention PCTs and control by cluster: 2009/10 to 2012/13**


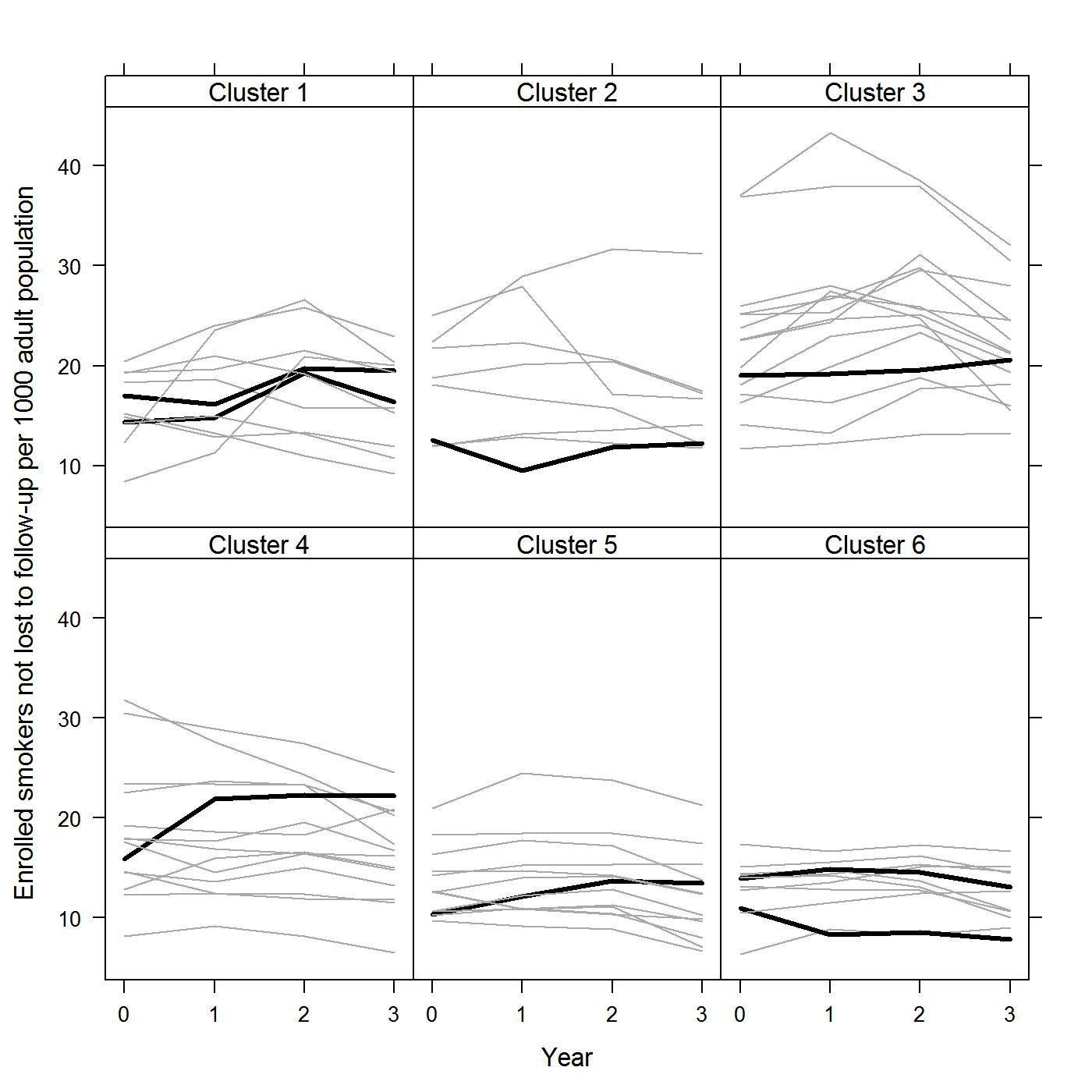

Supplement: S1 Fig — (DOCX) [file pone.0123349.s012.docx]
