# Supplementary material for: Effect of Pay-For-Outcomes and Encouraging New Providers on National Health Service Smoking Cessation Services in England: A Cluster Controlled Study
Source: PLoS One. 2015 Apr 15;10(4):e0123349. doi: 10.1371/journal.pone.0123349 (PMC4398496; doi:10.1371/journal.pone.0123349)
Supplement: S1 Text — (DOCX) [file pone.0123349.s015.docx]

**Supp****orting information**

**S1Text A measure of the impact of stop smoking services**

A measure of the reported impact of stop smoking services is the number of CO-validated four-week quits beyond those estimated to have occurred with only a prescription for smoking cessation medication and no behavioural support [9]. This measure estimates the contribution made by stop smoking services beyond that expected if the enrolled smokers had instead taken smoking cessation medication on prescription. The measure assumes that the stop smoking services had no effect on promoting quit attempts [9]. By 2012/13, this impact number of quits per 100,000 adult population was 378 in the intervention PCTs compared to 205 in the control PCTs (S10 Table). This finding provides further confirmation of the intervention’s performance. The measure deducts any shortfall in CO-verified quits experienced in the intervention PCTs below the 25% level from excess quits (above the 25% level) experienced in other invention PCTs when estimating the impact of the intervention as a whole. Alternatively, if we assume that the presence of stop smoking services in a locality with a quit rate of less than 25% have not caused the shortfall in quits, and so only count quits in excess of the 25% level, then the control PCTs’ impact increases to 225 quits per 100,000 adult population in 2012/13 (S11 Table).
