# Supplementary material for: Effect of Pay-For-Outcomes and Encouraging New Providers on National Health Service Smoking Cessation Services in England: A Cluster Controlled Study
Source: PLoS One. 2015 Apr 15;10(4):e0123349. doi: 10.1371/journal.pone.0123349 (PMC4398496; doi:10.1371/journal.pone.0123349)
Supplement: S2 Text — (DOCX) [file pone.0123349.s016.docx]

**Supp****orting information**

**S2 Text A note on cost-effectiveness**

From an NHS commissioner’s perspective, the maximum cost per quit in the intervention is the combined four-week and 12-week payments, which is £394 for general quitters and £641 for target quitters (S1 Table). These costs relate to 12-week quitters, and the relapse rate from 12 weeks to 12 months is uncertain. If we apply a 75% relapse rate associated with four-week quits, which may overestimate the relapse rate [1], the maximum cost per quit at 12 months is £1,576 for general quitters and £2,564 for target quitters. Quality-adjusted life-year (QALY) gains per 12-month quit have been estimated for general quitters to be 1.08 [2] and 1.29 [3]. These QALY gains may underestimate the gains for target quitters. However, applying a QALY gain per 12-month quit of 1.08 to the 12-month costs per quit, results in cost per QALY estimates of £1,459 for general quitters and £2,374 for target quitters. These estimates are well below the threshold for cost-effectiveness of £20,000 per QALY associated with NICE guidance.

References

1. Hughes J, Keely J, Naud S. Shape of the relapse curve and long-term abstinence among untreated smokers. Addiction. 2004;99: 29-38.

2. Woolacott N, Jones L, Forbes C, Mather L, Sowden A, Song F. et al. The clinical effectiveness and cost-effectiveness of bupropion and nicotine replacement therapy for smoking cessation: a systematic review and economic evaluation. Health Technol Assess. 2002;6(16): 1-245.

3. Cromwell J, Bartosch W, Fiore M, Hasselblad V, Baker T. Cost-effectiveness of the clinical practice recommendations in the AHCPR guideline for smoking cessation. JAMA. 1997;278: 1759-66.
